# Changes and backlogs in the provision and utilization of essential health services in Afghanistan during and after the COVID-19 pandemic

**DOI:** 10.1186/s12913-025-13093-x

**Published:** 2025-07-03

**Authors:** Narges Neyazi, Ali Mirzazadeh, Abdul Ghani Ibrahimi, Mirwais Ahmadzai, Jamshed Ali Tanoli

**Affiliations:** 1 Health System Development Department, World Health Organization, Kabul, Afghanistan; 2 Department of Epidemiology and Biostatistics, Institute for Global Health Sciences, University of California, San francisco, USA; 3 General Directorate for Policy and Planning, Ministry of Public Health, Kabul, Afghanistan; 4 Kabul University, Kabul, Afghanistan; 5 Country Representative, World Health Organization, Kabul, Afghanistan

**Keywords:** COVID-19 pandemic, Primary health care, Rural settings, Changes in utilization of health services, Afghanistan

## Abstract

**Introduction:**

Afghanistan is a low-income country where providing essential healthcare services is lifesaving for millions. The COVID-19 pandemic, the government and the international aid changes have affected the overburdened and fragile health system and put a risk on universal health coverage in Afghanistan. In this study, we aim to study the changes and backlogs to the essential health services during and after COVID-19 pandemic (Feb 2020 to Sep 2022) in Afghanistan.

**Method:**

A cross-sectional study of health facilities was conducted in nine provinces of Afghanistan. A randomly selected 165 public and private primary care centers and hospitals in 49 districts were studied. A WHO standardized questionnaire was used for this survey. Trained staff met the facility managers in person and completed the questionnaire by individual interviews during September 2022.

**Result:**

Hospitals located mostly in urban areas (*n* = 39, 65%) and clinics were located mostly in rural areas (*n* = 74, 71.1%) and governed by the government and the NGOs (76.6% of hospitals and 84.7% of clinics). The average number of staff per facility was 118 (SD = 180) for hospitals and 16(SD = 7) for clinics. 27 (46.5%) of hospitals and 44 (41.9%) of clinics reported that they experienced a higher outpatient service utilization in the previous month, compared to the month before. nearly half of the backlogs during the pandemic were related to routine preventive services such as annual check-ups antenatal care, and childhood immunization (55.0% for hospitals, and 45.7% for clinics). prioritizing high risk patients (86.7%), promoting self-care interventions wherever appropriate (75.0%), redirecting patients to alternative healthcare facilities (73.3%), providing all care in a single visit for multiple morbidities (60.0%), and providing home-based care for certain patients (58.3%) in hospitals. However, the most used strategies in clinics were prioritizing high risk patients (93.3%), redirecting patients to alternative healthcare facilities (75.2%), and providing home-based care for certain patients (66.7%).

**Conclusion:**

The pandemic exacerbated existing health inequities and hindered progress toward Universal Health Coverage (UHC). Health facilities employed various strategies to cope with the disruptions, such as prioritizing high-risk patients, promoting self-care, and redirecting patients to alternative facilities. However, the increased cost of transportation and health services, along with limited availability of medicines, remained significant barriers to healthcare access.

## Introduction

Afghanistan is a low-income country with a Human Development Index (HDI) value 0.478. In 2021, it ranked 180th among 191 countries and territories worldwide [1]. During the last two decades, significant progress has been made in coverage of essential health services in Afghanistan (an increase in coverage index from 23 in 2000 to 42 in 2019) [2]. The backbone of Afghanistan’s health system is Basic Package of Health Services and Essential Package of Hospital Services [3]. The Basic Package of Health Services (BPHS) started in 2003 and focuses on primary care, including maternal and newborn health, child health and immunization, public nutrition, communicable diseases, mental health, disability, and regular supply of essential drugs [4]. The second package, the Essential Package of Hospital Services (EPHS), started in 2005 and includes secondary and partially tertiary care in Afghanistan [5]. Many studies show significant progress toward service coverage and access to different essential health services within Afghanistan [6–10]. However, the COVID-19 pandemic has disrupted the provision and utilization of health services in many developed and developing countries [11].

The COVID-19 pandemic has affected many sectors, specifically fragile health systems in Low- and Middle-Income Countries (LMICs). It posed widespread challenges on all levels of health service delivery, especially during early times due to the steady spread of the virus within the continents [7]. As of July 2024, the World Health Organization (WHO) reported nearly 776 million confirmed cases and over seven million deaths globally from which over 23 million cases and 351 thousand deaths reported from the Eastern Mediterranean Region [12]. There are pieces of evidence that the COVID-19 pandemic has affected progress toward achieving Universal Health Coverage (UHC) globally [13–18]. However, how much the pandemic has affected the basic and essential health services in Afghanistan during its peak months has yet to be studied. Afghanistan experienced several waves (six main peaks) of COVID-19 cases from February 2020 to February 2023. The first major peak occurred in mid-2020, with a surge in cases and deaths reported in June and July 2020 [13]. The second peak was observed in December 2020- January 2021. However, the notable wave was in June-July 2021 (Delta variant), when the country recorded its highest daily number of cases [14]. The highest mortality rates were seen during these peaks, particularly affecting older adults and those with pre-existing conditions. The healthcare system, already strained by ongoing conflicts and limited resources, faced immense challenges in managing these surges [15]. Despite efforts to control the spread, including lockdowns and vaccination campaigns, the pandemic’s impact was profound, exacerbating existing health and socio-economic issues [16]. The fourth peak was during December 2021-January 2022 (Omicron variant), the fifth peak was observed in June 2022 and the sixth peak was in January 2023 [17, 18].

Despite imposing several restrictions on population movement and gathering and implementation of lockdowns, the country still experienced these waves mainly due to poor diagnostic and treatment capacity. The shortage of diagnostic kits, inadequate number of diagnostic centers, high cost of diagnosis in private sector, lack of kit production inside the country, shortage of trained laboratory staff and inadequate of safe and standard vehicles for transporting diagnostic kits are the main gaps. In addition, serious shortage of trained healthcare workers, facilities and equipment hindered provision of proper treatment for COVID-19 patients [19, 20].

In this national cross-sectional survey, we, we aim to explore the changes and backlogs in providing and utilizing essential health services during the pandemic (Feb 2020 to Sep 2022) in Afghanistan.

## Methods

**Fig. 1:**
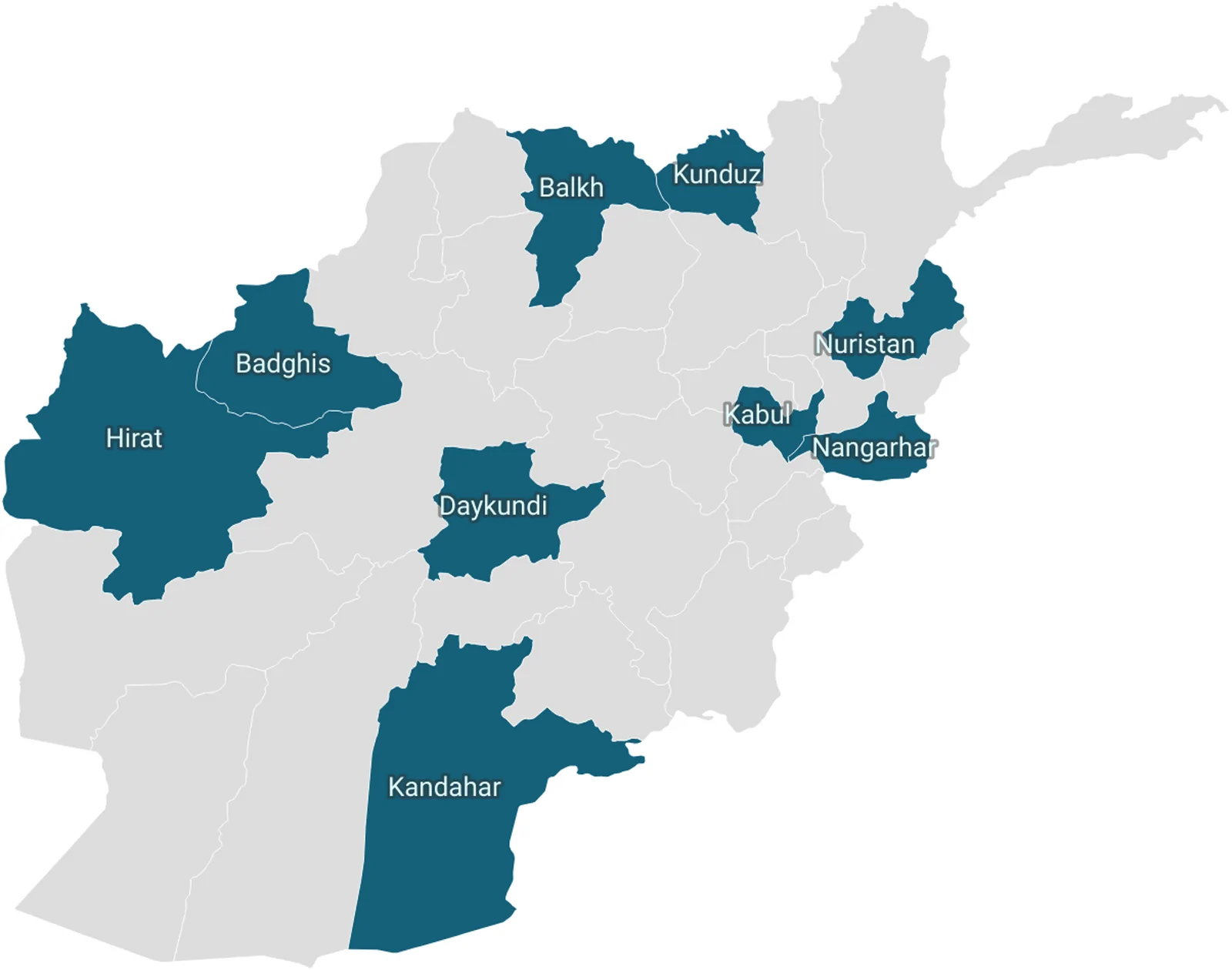
Selected provinces in Afghanistan for the survey

We conducted a cross-sectional study of health facilities in nine provinces of Afghanistan, including Kabul, Nangarhar, Herat, Kandahar, Balkh, Daykondi, Nooristan, Kunduz, and Badghis (Fig. 1). Altogether, these nine provinces included 49% of the total population in 2020 [21]. The tool, service readiness for COVID-19 case management and essential health service provision [22, 23], can be used by countries to rapidly assess the capacity of health services during the pandemic. The tool aims to help alert the authorities and other stakeholders about where provision and utilization of COVID-19 and essential health services may require modification and/or investment. It can be used once to provide a rapid snapshot of status, or on a regular basis for tracking and monitoring the service readiness during the different phases of the pandemic. In Afghanistan, it is the second round of this survey, the first round was implemented in five zone provinces (Kabul, Nangarhar, Kandahar, Herat, Balkh) during the February 2022. The questionnaire for this survey was developed by the WHO Headquarters and modified for the local setting of the Afghanistan health system by the WHO Afghanistan country office. Then, the questionnaire was validated by national experts in a national workshop in January 2022. Before the first round of the survey, the tool was piloted. The questionnaire was not translated into local language, as the interviewers were proficient in both English and local languages (Dari and Pashto).

We used two-stage random sampling to select a representative sample of healthcare centers and hospitals in the nine provinces. In first stage, we randomly selected 49 (out of 130) districts in these nine provinces using PPS (Probability-Proportional-to-Size Sampling). To select health facilities, we used a comprehensive sample frame of public facilities with exact location addresses provided by Ministry of Public health. Unfortunately, there was not such sampling framework for private health facilities. So, we included all private health facilities located in the selected districts of the province. We obtained the list and address of these private health facilities from Provincial Public Health Department (PPHDs) of these provinces. Having a combined framework for public and private health facilities, 165 (out of 300) primary public health care centers and hospitals randomly were selected in the selected districts. We excluded facilities that were specifically designated to provide COVID-19 care at the time of study. However, one question was about if the facility has been ever designated to COVID-19 care during the pandemic. Data collection was completed from 14 to 23 September 2022 simultaneously in all selected provinces, and the facility managers were asked to report on disruptions in service provision and changes in service delivery strategies.

Trained staff met in person with the facility managers and completed the questionnaire by individual interviews during September 2022. The responses were entered electronically using the offline Lime Survey application [24] and uploaded to a secure database. The questionnaire had 253 questions and took an average of 80 min to complete. In this paper, we present the findings for the following sections: staffing, and delivery and utilization of essential health services, which includes 59 questions.

Data were exported into Excel, cleaned the raw data by removing inconsistencies, repetition, and data entry errors, and then exported into and analyzed using STATA version 17. We calculated the frequency distribution of characteristics of facilities and responses to questions.

The study protocol was reviewed and approved by the Ministry of Public Health institutional review board (IRB Code No: A.0122.0389). Verbal informed consent was obtained from each facility manager who was interviewed. They were told that participation is voluntary, and they can leave the interview any time they would like.

## Results

We enrolled managers from 165 public and private health facilities in nine provinces of Afghanistan. We analyzed the data disaggregated by type of health facilities (hospital and health clinics). The majority of health clinics were in rural areas (*n* = 74, 71.1%), while 35 (65%) of hospitals located in urban setting. Most of health facilities was managed by government/NGOs (*n* = 46, 76.6% of hospitals and *n* = 89, 84.7% of clinics). 17 (28.3%) hospitals were designated as a COVID-19 center during the pandemic. The average number of medical doctors, nurse, and midwives are (24.6, 25.5, and 7.7 respectively) for hospitals. An average of 105 (89%) out of 118 averaged total staff received at least one dose of COVID-19 vaccine in hospitals and an average of 13(81.0%) out of 15.7 total averaged number of staff received one dose of COVID-19 vaccines in clinics. 38 (63.3%) hospitals and 60 (57.1%) clinics received training for COVID-19 topics in the previous year (Table 1).

**Table 1:** Characteristics of health care facilities assessed in Sep 2022, Afghanistan (*N* = 165)

Characteristics	Hospitals (*N* = 60)		Clinics (*N* = 105)	
	*n*	%	*n*	%
Facility Location				
Rural	21	35	74	71.1
Urban	39	65	30	28.8
Managing authority				
Government/NGO	46	76.6	89	84.7
Private for profit	14	23.3	16	15.2
A designated COVID-19 center during the pandemic				
Yes	17	28.3	4	3.8
staffing, new infection, vaccination				
Average number of staff per facility (Mean, SD)	118.0	180.0	16.0	7
Total number of medical doctor (Mean, SD)	24.6	54.2	1.3	1.5
Total number of nurse (Mean, SD)	25.5	54.3	1.5	1.2
Total number of midwives (Mean, SD)	7.7	14.7	1.4	0.8
Average number of staff who received COVID-19 vaccine (at least one dose)	105	89.0	13	81.2
Staff who were on leave or absent in the previous one week due to reasons related to COVID-19 (Mean, SD)	1.1	2.1	0.1	0.4
Training and supportive supervision for COVID-19 topics				
Facilities received training related to COVID-19 for their staff in the previous one year	38.0	63.3	60	57.1
Proper use of personal protective equipment (PPE)	37.0	97.4	46	78
Environmental cleaning and disinfection	36	94.7	51	85
Screening of COVID-19 signs and symptoms at the point of first presentation	29	78.4	46	76.7
Hand hygiene	33	86.8	52	88.1
Prioritizing patients on arrival using a triage tool	34	89.5	45	75
Management of emergency conditions such as difficulty in breathing, shock, and altered mental status	31	83.8	36	60
Provision of remote healthcare such as telemedicine	22	57.9	29	48.3
Facilities received supportive supervision related to COVID-19 in the previous one year	29	48.3	60	57.1

### Outpatient service utilization

27 (46.5%) of hospitals and 44 (41.9%) of clinics reported that they experienced a higher overall outpatient service utilization in the previous month, compared to the month before. The factors that may be associated with the higher number of outpatient visits are typical increase in other infectious disease because of changing seasons or weather (28.3%), more patients presenting with acute respiratory infection symptoms (21.7%), more patients being redirected from other facilities (18.3%), and general health communication campaigns to promote care seeking (10.0%) for hospitals. For clinics, the main reasons were seasonal increase in other infectious diseases (31.4%), more patients presenting with acute respiratory infection symptoms (18.1%), and general health communication campaign to promote care-seeking (12.4%). Potential factors for the lower number of OPD visits were increased cost for transportation (11.7%) and increased cost for health services (10.0%) for hospitals. While the main reason for clinics was seasonal decrease in other infectious diseases because of changing seasons or weather (14.3%), changes in recommendation to the public for mild illness and elective care (7.6%) and limited availability of medicines or consumables (7.6%) in the clinics (Table 2).

**Table 2:** Changes in utilization and provision of essential health services and its reason in assessed health facilities, Sep 2022, Afghanistan (*N* = 165)

Changes	Hospitals (*N* = 60)		Clinics (*N* = 105)	
	*n*	%	*n*	%
Outpatient service utilization				
Higher	27.0	46.5	44	41.9
Similar	16.0	27.6	37	35.2
Lower	15.0	25.8	24	22.8
*Potential reasons for the lower number of outpatient visits in the previous one month*,* compared to the month before*				
Decrease in COVID-19 transmission	3	5.0	7	6.6
Typical decrease in other infectious diseases because of changing seasons or weather	5	8.3	15	14.3
Changes in recommendation to the public for mild illness and elective care	3	5.0	8	7.6
Fear, mistrust, uncertainty about catching COVID-19 during facility visits	1	1.7	0	0
Lockdown or stay at home order	2	3.3	6	5.7
Disruption of public transport	2	3.3	6	5.7
Increased cost for transportation	7	11.7	5	4.7
Scope of specific services reduced	1	1.7	2	1.9
Provision of specific services completely suspended	0	0.0	0	0
Reduced or changed opening hours	1	1.7	1	0.9
Facility closure	1	1.7	1	0.9
Limited availability of medicines or consumables	0	0.0	8	7.6
Limited availability of medical staff	0	0.0	2	1.9
Long waiting hours at facility	4	6.7	1	0.9
Increased cost for health services	6	10.0	4	3.8
*Potential reasons for the higher number of outpatient visits in the previous one month*,* compared to the month before*				
More patients presenting with acute respiratory infection symptoms	13	21.7	19	18.1
Typical increase in other infectious diseases because of changing seasons or weather	17	28.3	33	31.4
More clients presenting with GBV related issues	2	3.3	3	2.8
More patients being redirected from other facilities	11	18.3	5	4.7
Backlog from disruptions of services prior to the past month	2	3.3	3	2.8
Communication to the public about reactivation of any services that were previously suspended or reduced	3	5.0	6	5.7
General health communications campaign to promote care-seeking	6	10.0	13	12.4
*Community outreach or home visits*				
Immunization outreach (*n* = 89)				
Higher	7	11.7	18	17.1
Similar	2	3.3	22	20.9
Lower	13	21.7	29	27.6
Malaria prevention campaigns, including distribution of insecticide-treated nets (*n* = 55)				
Higher	1	1.6	4	3.8
Similar	9	15.0	42	40
Lower	13	21.6	23	21.9
Neglected tropical disease outreach activities, including mass drug administration (*n* = 56)				
Higher	1	1.6	5	4.7
Similar	9	15	45	42.8
Lower	13	21.6	19	18.1
Community -based mobile clinics (*n* = 51)				
Higher	4	6.6	8	7.6
Similar	6	10.0	45	42.8
Lower	13	21.6	16	15.2
Home visits (*n* = 86)				
Higher	5	8.3	18	17.1
Similar	5	8.3	23	21.9
Lower	13	21.6	28	26.6

### Community outreach or home visits

Facility mangers were asked about changes in frequency of outreach services in the previous month, compared to the same one month last year. Most facilities reported a decrease in immunization outreach (13 (21.7%) of hospitals and 29 (27.6%) of clinics), malaria prevention campaigns (13 (21.6%) of hospitals and 23(21.9%) of clinics), neglected tropical disease outreach activities (13 (21.6%) of hospitals), community-based mobile clinics (13 (21.6%) of hospitals, and home visits (13 (21.6%) of hospitals, and 28 (26.6%) of clinics). However, 45 (42.8%) of clinics reported similar activities for neglected tropical disease outreach activities, as well as community-based mobile clinics (*n* = 45,42.8%) (Table 2).

Table 3 presented backlog per services in assessed health facilities. We defined backlog for interviewees as following: “During the COVID-19 pandemic, sometimes, facilities have not been able to provide certain outpatient services as usual. In some facilities, that have resulted in backlogs of patients who wait for their turns. Since the start of the pandemic, has this facility ever had backlogs related to the COVID-19 pandemic in the following type of services?”. As presented (Table 3), nearly half of the backlogs during the pandemic were related to routine preventive services such as annual check-ups antenatal care, and childhood immunization (55.0% for hospitals, and 45.7% for clinics). In addition to this, the most backlogged services during the pandemic were those for non-communicable diseases (43.3% for hospitals and 44.7% for clinics), and TB and HIV/AIDS (41.7% for hospitals, and 37.1% for clinics).

**Table 3:** Backlog per services in assessed health facilities during the pandemic, Sep 2022, Afghanistan (*N* = 165)

Backlog per services	Hospitals (*N* = 60)		Clinics (*N* = 105)	
	*n*	%	*n*	%
During the pandemic				
Routine preventive services such as annual check-ups, antenatal care, childhood immunization	33	55.0	48	45.7
Management of non-communicable diseases	26	43.3	47	44.7
Management of infectious diseases such as TB and HIV/AIDS	25	41.7	39	37.1
Elective surgeries or procedures	26	43.3	14	13.3
During last month (Aug-Sep 2022)				
Routine preventive services such as annual check-ups, antenatal care, childhood immunization	29	48.3	40	38.1
Management of non-communicable diseases	23	38.3	41	39
Management of infectious diseases such as TB and HIV/AIDS	23	38.3	35	33.3
Elective surgeries or procedures	24	40	15	14.3

Table 4 shows the strategies used by the health facilities for safe and continued delivery of services during the pandemic. The interviewees were asked the following question: “now I am going to ask you about services that are unrelated to COVID-19. During the pandemic, some facilities have provided services differently at times. Has the facility done any of the following ever during the pandemic?.

These strategies were prioritizing high risk patients (86.7%), promoting self-care interventions wherever appropriate (75.0%), redirecting patients to alternative healthcare facilities (73.3%), providing all care in a single visit for multiple morbidities (60.0%), and providing home-based care for certain patients (58.3%) in hospitals. However, the most used strategies in clinics were prioritizing high risk patients (93.3%), redirecting patients to alternative healthcare facilities (75.2%), and providing home-based care for certain patients (66.7%).

**Table 4:** Strategies used for safe and continued delivery of services during the pandemic, Sep 2022, Afghanistan (*N* = 165)

Strategies	Hospitals (*N* = 60)		Clinics (*N* = 105)	
	*n*	%	*n*	%
Reduced the scope of specific services	31	51.6	53	50.5
Reduced the volume of specific services	17	28.3	24	22.9
Suspended the provision of specific services	25	41.7	36	34.3
Redirected patients to alternative healthcare facilities	44	73.3	79	75.2
Given priority to seeing high-risk patients	52	86.7	98	93.3
Provided all care in a single visit for multiple morbidities	36	60.0	66	62.9
Supported self-care interventions wherever appropriate	45	75.0	84	80
Provided home-based care for certain patients	35	58.3	70	66.7
Shifted clinical encounters to digital platforms such as teleconsultations	29	48.3	39	37.1
Provided electronic or tele prescriptions	27	45.0	40	38.1
Extended prescriptions of medicines for long-term use, such as medicines for treating non-communicable diseases	38	63.3	50	47.6
Used novel dispensing approaches for medicines	30	50.0	44	41.9

## Discussion

In our study, most of hospitals located in urban setting and managed by government/NGOs. At the time of the survey, we found that nearly one third of hospitals were repurposed as COVID-19 center during the pandemic. More than half of the hospitals and clinics received training for COVID-19 topics in the previous year and almost 90% of staff in hospitals and clinics received at least one dose of COVID-19 vaccine. Health facilities experienced a higher level of outpatient service utilization after the pandemic peak, mainly due to seasonal non-COVID-19 infections (other infectious diseases that are diagnosed at facility level) and more frequent acute respiratory infection symptoms. Meanwhile, the main reason for the lower number of OPD visits at hospitals were increased transportation cost and increased cost for health services, while the main reason for lower number of OPD visits were decrease in seasonal infectious diseases, changes in recommendation to the public for mild illness and elective care and limited availability of medicine or consumables in the clinics. However, most health facilities reported a decrease in community-based outreach programs. Health facilities used strategies such as prioritizing high-risk patients, promoting self-care interventions, and redirecting patients to other health facilities to respond to these fluctuations.

The COVID-19 pandemic disrupted the delivery of essential healthcare services worldwide. This effect was worse in countries with fragile health systems and existing health inequities. The pandemic also affected the achievement of universal access to healthcare and provision of equal opportunities for healthcare utilization for all populations [25]. As a global movement toward Universal Health Coverage (UHC), Sustainable Development Goal (SDG) 3.8 target focuses on this concept. This target is measured using two metrics: SDG indicator 3.8.1 on the coverage of essential health services and SDG indicator 3.8.2 on catastrophic health spending.

In this study, we found that the most reported backlogs were for routine essential health services such as antenatal care, childhood immunization, and management of communicable and non-communicable diseases. The backlogs were reported higher in hospitals comparing to clinics. It may be due to not considering the referral pathway by Afghan population, as they directly seek diagnosis and treatment services from hospitals. The results of a study conducted jointly by WHO/Europe, the European Observatory on Health Systems and Policies, and the Nuffield Trust in 2022 show that backlog and delays in non-emergency health care have stretched the health system and left many people without care [26]. A study in Iran showed that after an initial significant reduction of hospitalization rate, emergency department visits and outpatient visits in the first month of COVID-19 outbreak, significant monthly increases were observed in these three areas during the COVID-19 pandemic [27].

Many health facilities in Afghanistan implemented several strategies to maintain the delivery of their essential services during the COVID-19 pandemic. These strategies were prioritizing high-risk patients, redirecting patients to alternative healthcare facilities, and providing home-based care to certain patients in both hospitals and clinics and promoting self-care in both hospitals and clinics. While we have yet to find any study on the effectiveness of these strategies, we recommend program managers and service providers assess the efficacy of these strategies at the facility, provincial, and national levels. A study in India showed that to maintain service provision, home-based services increased, and use of PPE, telephones for communication and applying social distancing have been increased [28].

Responding to backlogs, especially for essential health services, and delivering alternative strategies to maintain the health services must strengthen the health system focusing on the health workforce. Coates et al. (2021) framework suggested three types of interventions to increase health workforce capacity during a pandemic: (1) increase the numbers of health workers; (2) improve the skills of the health workforce by increasing the scope of work and their flexibility; (3) increase the support required in terms of medicine and supply to increase the sustainability of service provision [29]. Some of good initiatives to train and deploy health workforce during the pandemic in Afghanistan include training doctors and nurses in Rapid Response Teams (RRTs) and Contact Tracing teams and deploying them to the borders to detect and treat the suspected cases, train and deploy home-based care teams (a team of one doctor and one nurse) to provide diagnosis and treatment for mild and moderate cases of COVID-19 at their homes, training lab technicians on COVID-19 rapid and PCR tests and deploying them to 6 regional lab centers across the country, establishing COVID-19 treatment centers and training the health workforce on using PPE, social distancing, infection prevention, diagnosis and treatment of COVID-19 patients [30, 31]. In 2023, Afghanistan has a density of 10.3 doctors, nurses, and midwives per 10,000 population [32]. It was much lower than the minimum threshold density recommended by WHO, which is 44.5 doctors, nurses, and midwives per 10,000 [33]. Afghanistan needs to train more students to become doctors, nurses, or midwives to cover this gap in its workforce. Training more women to hold doctor, nurse and midwifery jobs is critical for Afghanistan’s health system, considering the cultural barriers in Afghan community. This should consider geographical balance throughout the country as well.

In addition, we found that health facilities decreased their community outreach healthcare services during the pandemic. Other studies also showed challenges in providing community-based healthcare services during the COVID-19 pandemic [34, 35]. One alternative could be promoting self-care among the population. Self-care can be used for the prevention or management of non-communicable and communicable diseases, mental health, and sexual and reproductive health [36–38].

One of the main findings of our study is that the main potential reason for the low utilization of health services is the increased cost of transportation and health services at hospital level. Although there is evidence of high Out-of-pocket payments in Afghanistan, more than 70% during the recent decade [39], this finding is also worth considering. The need for a sustainable health financing system aimed at decreasing the OOP and catastrophic cost of healthcare has always been a concern for Afghanistan [40] which was never achieved due to political instability in this country. The regime changes in 2021 and suspension of donor funding to the health system deteriorated the situation more [41]. The findings of our study also showed that one of the main reasons of low utilization of health services at clinic level was limited availability of medicines and consumables. The findings from different studies especially after 2021 are consistent with the result of our study [42, 43]. A qualitative study during 2022 using in-depth interviews with 43 health workers in ten provinces of Afghanistan also showed that the main barriers to achieving UHC in Afghanistan are severe shortages of facilities, equipment, workers and medications at facility levels especially at rural areas. The study also highlighted that the quality of available medications is under question too [44]. A study in Pakistan also showed that the main barriers to care-seeking were stockouts and lack of supplies at facility level [45].

Our study had five main limitations. First, our data on changes in the services were based on the survey with health facility managers, but not on the actual utilization record. So, it includes first, recall bias in terms of response of facility managers about service delivery for the past month or the past year. Second, as the facility managers were not the actual beneficiary of the services with real experience, they might not provide a response based on what was real in health facilities. The third limitation is that we did not collect patients’ perspectives and reasons for less access to health services during the COVID-19 pandemic and we presented the reasons based on the responses of facility managers’ perception which can be differ from patients’ perspective. To our knowledge, we could not find any population or patient- based study focusing on service delivery assessment during the pandemic. Fourth, under the scope of our study, we could not explore the influence of reduced community activities on preventive care such as vaccination or curative care for communicable and non-communicable diseases. Future qualitative studies are recommended to explore this aspect.

fifth, our study did not examine the link between using the coping mechanisms and the increased service utilization rate. In addition, we did not explore whether the coping mechanisms have been applied to the period of COVID outbreak or rather for period before and after the pandemic. We recommend future studies to explore any possible association in health facilities.

## Conclusion

The COVID-19 pandemic has significantly disrupted essential healthcare services worldwide, particularly in countries with fragile health systems like Afghanistan. Our study highlights the challenges faced by health facilities, including increased outpatient service utilization due to non-COVID-19 infections and decreased community-based outreach programs. The pandemic exacerbated existing health inequities and hindered progress toward Universal Health Coverage (UHC). Health facilities employed various strategies to cope with the disruptions, such as prioritizing high-risk patients, promoting self-care, and redirecting patients to alternative facilities. However, the increased cost of transportation and health services, along with limited availability of medicines, remained significant barriers to healthcare access. The study underscores the need for sustainable health financing, training more healthcare workers, especially women, and assessing the effectiveness of coping strategies. Future studies should explore patients’ perspectives and the impact of reduced community activities on preventive care. Addressing these challenges is crucial for strengthening Afghanistan’s health system and achieving UHC. International donors are recommended to support establishing a resilient health system with sustainable financing mechanism in Afghanistan. Due to two main limitations of our study, we recommend further studies on the effectiveness of coping mechanisms on maintaining provision of essential health services and utilization of essential health services based on facility records and patient perspective.

## Data Availability

The datasets generated and analyzed during the current study are not publicly available as the informed consent applied only for the use by the research team. If desired, the data can be viewed and reviewed together with the corresponding author.

## Abbreviations

HDI: Human Development Index
BPHS: Basic Package of Health Services
EPHS: Essential Package of Hospital Services
LMICs: Low ad Middle Income Countries
WHO: World Health Organization
UHC: Universal Health Coverage
OPD: Outpatient Department
TB: Tuberculosis
HIV: Human Immune deficiency Virus
AIDS: Acquired Immunodeficiency Syndrome

## References

[1] United Nations for Development Program. Human Development Reports 2021. https://hdr.undp.org/data-center/country-insights#/ranks. Accessed 01 Nov 2023.

[2] The World Bank. SDG 3.8.1 Coverage of essential health service. https://databank.worldbank.org/embed/SDG-3.8.1-Coverage-of-essential-health-services/id/f609d4b. Accessed 01 Nov 2023.

[3] World Health Organization. Afghanistan Health System. https://www.emro.who.int/afg/programmes/health-system-strengthening.html#:~:text=Afghanistan’s%20health%20system%20has%20been,population%20within%20two%20hours%20distance. Accessed 01 Nov 2023.

[4] Newbrander W, Ickx P, Feroz F, Stanekzai H. Afghanistan’s basic package of health services: its development and effects on rebuilding the health system. Glob Public Health. 2014. 10.1080/17441692.2014.916735. PMC4136668 24865404

[5] Afghanistan Ministry of Public health. Essential Package of Hospital Services 2005. https://platform.who.int/docs/default-source/mca-documents/policy-documents/guideline/afg-cc-46-01-guideline-2005-eng-essential-hospital-services.pdf. Accessed 01 Nov 2023.

[6] Kim YM, Zainullah P, Mungia J, Tappis H, Bartlett L, Zaka N. Availability and quality of emergency obstetric and neonatal care services in Afghanistan. Int J Gynaecol Obstet. 2012. 10.1016/j.ijgo.2011.10.017. 22196990

[7] Edward A, Branchini C, Aitken I, Roach M, Osei-Bonsu K, Arwal SH. Toward universal coverage in afghanistan: A multi-stakeholder assessment of capacity investments in the community health worker system. Soc Sci Med. 2015. 10.1016/j.socscimed.2015.06.011. 26141453

[8] Akseer N, Bhatti Z, Rizvi A, Salehi AS, Mashal T, Bhutta ZA. Coverage and inequalities in maternal and child health interventions in Afghanistan. BMC Public Health. 2016. 10.1186/s12889-016-3406-1. PMC5025831 27634540

[9] Mbaeyi C, Kamawal NS, Porter KA, Azizi AK, Sadaat I, Hadler S, Ehrhardt D. Routine immunization service delivery through the basic package of health services program in afghanistan: gaps, challenges, and opportunities. J Infect Dis. 2017. 10.1093/infdis/jiw549. PMC5853859 28838158

[10] Howard N, Woodward A, Patel D, Shafi A, Oddy L, Veen A, et al. Perspectives on reproductive healthcare delivered through a basic package of health services in afghanistan: a qualitative study. BMC Health Serv Res. 2014. 10.1186/1472-6963-14-359. PMC4169831 25167872

[11] World Health Organization. Third round of the global pulse survey on continuity of essential health services during the COVID-19 pandemic. 2021. https://www.who.int/publications/i/item/WHO-2019-nCoV-EHS_continuity-survey-2022.1. Accessed 02 Nov 2023.

[12] World Health Organization. WHO COVID-19 dashboard. https://data.who.int/dashboards/covid19/deaths?n=o&m49=957. Accessed 13 Aug 2024.

[13] Haileamlak A. The impact of COVID-19 on health and health systems. Ethiop J Health Sci. 2021. 10.4314/ejhs.v31i6.1. PMC8968362 35392335

[14] Saengtabtim K, Tang J, Leelawat N, Egawa S, Suppasri A, Imamura F. Universal health coverage mitigated COVID-19 health-related consequences in Asia Oceania. Int J Disaster Risk Reduct. 2023. 10.1016/j.ijdrr.2023.103725. PMC10141793 37193307

[15] Mao W, Ogbuoji O, Watkins D, Bharali I, Nsiah-Boateng E, Diab MM, et al. Achieving global mortality reduction targets and universal health coverage: the impact of COVID-19. PLoS Med. 2021. 10.1371/journal.pmed.1003675. PMC8270396 34166391

[16] Al-Awlaqi S, Dureab F, Annuzaili D, Al-Dheeb N. COVID-19 in conflict: the devastating impact of withdrawing humanitarian support on universal health coverage in Yemen. Public Health Pract (Oxf). 2020. 10.1016/j.puhip.2020.100015. PMC7250073 34171044

[17] Nguyen Thu H, Nguyen Quynh A, Khuat Hai O, Le Thi Thanh H, Nguyen Thanh H. Impact of the COVID-19 pandemic on provision of HIV/AIDS services for key populations. Int J Health Plann Manage. 2022. 10.1002/hpm.3508. PMC9348422 35607717

[18] Cuschieri S, Grech E, Gatt A, Cutajar A, Vassallo C, Zahra D, et al. The impact of the COVID-19 pandemic on the mediterranean region over 18 months: bridging the health outcomes and sustainable development goals. Afr Health Sci. 2022. 10.4314/ahs.v22i4.61. PMC10126880 37113533

[19] Mousavi SH, Abdi M, Zahid SU, Wardak K. Coronavirus disease 2019 (COVID-19) outbreak in afghanistan: measures and challenges. Infect Control Hosp Epidemiol. 2021;42(3):366–7. 10.1017/ice.2020.240. PMC7264446 32412402

[20] Essar MY, Hasan MM, Islam Z, Aeel Riaz MM, Aborode AT, Ahmad Sh. COVID-19 and multiple crises in afghanistan: an urgent battle. Confl Health 2021; 15(70). 10.1186/s13031-021-00406-0. PMC8447801 34535160

[21] Amimo F, Lambert B, Magit A, Hashizume M. A review of prospective pathways and impacts of COVID-19 on the accessibility, safety, quality, and affordability of essential medicines and vaccines for universal health coverage in Africa. Global Health. 2021. 10.1186/s12992-021-00666-8. PMC8027968 33832487

[22] The World Bank. Afghanistan Province Dashboard. https://www.worldbank.org/en/data/interactive/2019/08/01/afghanistan-interactive-province-level-visualization. Accessed 03 Nov 2023.

[23] World Health Organization. Monitoring frontline service readiness capacities during the COVID-19 pandemic. https://www.who.int/teams/integrated-health-services/health-services-performance-assessment/monitoring-health-services/monitoring-frontline-service-readiness-capacities-during-the-covid-19-pandemic. Accessed 03 Nov 2023.

[24] Neyazi N, Lindan C, Perdes S, Ibrahimi AG, Horemans D, Al Afsoor D. The provision and utilization of essential health services in Afghanistan during COVID-19 pandemic. Front Public Health. 2023. 10.3389/fpubh.2022.1097680. PMC9878336 36711388

[25] Limesurvey Organization. English Support Forum. https://forums.limesurvey.org/forum/can-i-do-this-with-limesurvey/59814-offline-surveys. Accessed 03 Nov 2023.

[26] Hussain R, Arif S. Universal health coverage and COVID-19: recent developments and implications. J Pharm Policy Pract. 2021. 10.1186/s40545-021-00306-x. PMC7874562 33568229

[27] Mahmoodpour-Azari M, Rezaei S, Badiee N, Hajizadeh M, Mohammadi A, Kazemi-Karyani A, et al. The COVID-19 pandemic and healthcare utilization in iran: evidence from an interrupted time series analysis. Osong Public Health Res Perspect. 2023;14(3):180–7. 10.24171/j.phrp.2023.0041. PMC10522821 37415435

[28] Phuong HN, Shivani K, Anjali P, Lan MT, Monika W, Sebanti G, et al. COVID-19 disrupted provision and utilization of health and nutrition services in Uttar pradesh, india: insights from service providers, Houshold phone surveys, and administrative data. J Nutr. 2021;151(8):2305–16. 10.1093/jn/nxab135. PMC8195077 34236434

[29] European observatory on health systems and policies. Addressing backlogs and managing waiting lists during and beyond the COVID-19 pandemic. 2022. https://www.who.int/europe/news/item/20-07-2022-covid-19-has-caused-major-disruptions-and-backlogs-in-health-care--new-who-study-finds. Accessed 20 Nov 2023. 36800878

[30] Lucero-Prisno DE 3rd, Ahmadi A, Essar MY, Lin X, Adebisi YA. Addressing COVID-19 in afghanistan: what are the efforts and challenges? J Glob Health. 2020;10(2):020341. 10.7189/jogh.10.020341. PMC7568006 33110541

[31] Yen MY, Schwartz J, King CC, Lee CM, Hsueh PR. Society of Taiwan Long-term care infection prevention and control. Recommendations for protecting against and mitigating the COVID-19 pandemic in long-term care facilities. J Microbiol Immunol Infect. 2020;53(3):447–53. 10.1016/j.jmii.2020.04.003. PMC7194976 32303480

[32] Coates A, Fuad AO, Hodgson A, Bourgeault IL. Health workforce strategies in response to major health events: a rapid scoping review with lessons learned for the response to the COVID-19 pandemic. Hum Resour Health. 2021. 10.1186/s12960-021-00698-6. PMC8685817 34930337

[33] Neyazi N, Yaghmaei N, Ahmadzai M, Kleipool E, Naumann N, Wassenaar M, Omar MH, Gedik FG, Alba S, Dieleman M, Ibrahimi AG, AbouZeid A. Assessing the health workforce in Afghanistan: a situational analysis into the country’s capacity for Universal health coverage. Confl Health. 2025;19(1):25. 10.1186/s13031-025-00663-3. PMC12007245 40247368

[34] World Health Organization. Global strategy on human resources for health: workforce 2030. https://www.who.int/publications/i/item/9789241511131. Accessed 30 Nov 2023.

[35] O’Sullivan B. Challenges and innovations in access to community-based rural primary care services during the Covid-19 pandemic in Australia. Int J Health Plann Manage. 2022. 10.1002/hpm.3598. PMC9878203 36443892

[36] Mahoney KJ. Self-Direction of home and Community-Based services in the time of COVID-19. J Gerontol Soc Work. 2020. 10.1080/01634372.2020.1774833. 32501150

[37] Narasimhan M, Aujla M, Van Lerberghe W. Self-care interventions and practices as essential approaches to strengthening health-care delivery. Lancet Glob Health. 2023. 10.1016/S2214-109X(22)00451-X. PMC9597558 36306809

[38] Patel V, Chisholm D, Parikh R, Charlson FJ, Degenhardt L, Dua T, et al. Addressing the burden of mental, neurological, and substance use disorders: key messages from disease control priorities, 3rd edition. Lancet. 2016. 10.1016/S0140-6736(15)00390-6. 26454360

[39] Riegel B, Moser DK, Buck HG, Dickson VV, Dunbar SB, Lee CS, et al. Self-Care for the prevention and management of cardiovascular disease and stroke: A scientific statement for healthcare professionals from the American heart association. J Am Heart Assoc. 2017. 10.1161/JAHA.117.006997. PMC5634314 28860232

[40] Afghanistan Ministry of Public Health. National Health Account report. 2019. https://extranet.who.int/countryplanningcycles/sites/default/files/planning_cycle_repository/afghanistan/afghanistan_national_health_accounts.pdf. Accessed 24 Nov 2023.

[41] Higgins-Steele A, Farewar F, Ahmad F, Qadir A, Edmond K. Towards universal health coverage and sustainable financing in afghanistan: progress and challenges. J Glob Health. 2018. 10.7189/jogh.08.020308. PMC6207102 30410734

[42] Safi N, Anwari P, Safi H. Afghanistan’s health system under the Taliban: key challenges. The Lancet. 22;400(10359),1179–1180. 10.1016/S0140-6736(22)01806-2 36162414

[43] Roien R, Essar MY, Ahmadi A, Lucero-Prisno Iii DE, Yousefi AA, Hasan MM, et al. Challenges of drug supply: how Afghanistan is struggling. Public Health Pract (Oxf). 2021;2:100129. 10.1016/j.puhip.2021.100129. PMC9461608 36101627

[44] Lamberti-Castronuovo A, Valente M, Bocchini F, Trentin M, Paschetto M, Bahdori GA, et al. Exploring barriers to access to care following the 2021 socio-political changes in afghanistan: a qualitative study. Confl Health. 2024;18(1):36. 10.1186/s13031-024-00595-4. PMC11044283 38658962

[45] Paudel M, Leghari A, Ahmad AM, Gibbs S, Wheeler J, Goldberg S, et al. Understanding changes made to reproductive, maternal, newborn and child health services in Pakistan during the COVID-19 pandemic: a qualitative study. Sex Reproductive Health Matters. 2022;30(1). 10.1080/26410397.2022.2080167. PMC9310789 35867009

