# Correction: Changes and backlogs in the provision and utilization of essential health services in Afghanistan during and after the COVID-19 pandemic

**DOI:** 10.1186/s12913-026-14229-3

**Published:** 2026-08-10

**Authors:** Narges Neyazi, Ali Mirzazadeh, Abdul Ghani Ibrahimi, Mirwais Ahmadzai, Jamshed Ali Tanoli

**Affiliations:** 1 Health System Development Department, World Health Organization, Kabul, Afghanistan; 2 Department of Epidemiology and Biostatistics, Institute for Global Health Sciences, University of California, San francisco, USA; 3 General Directorate for Policy and Planning, Ministry of Public Health, Kabul, Afghanistan; 4 Kabul University, Kabul, Afghanistan; 5 Country Representative, World Health Organization, Kabul, Afghanistan

**Correction: BMC Health Services Research (2025) 25:923**

10.1186/s12913-025-13093-x

In the original version of this article, the given and family names of [Ahmad Mirwais Ahmadzai] were incorrectly structured. The name was displayed correctly in all versions at the time of publication.

The original article has been corrected with the correct author name Mirwais Ahmadzai.

